# The No Stamp and Sample Copy Man

**Published:** 1878-06-20

**Authors:** 


### THE NO STAMP AND SAMPLE COPY MAN.

To the following ode from the *Courier-Journal* to the *no stamp man*, we add an additional verse to the *sample copy man*, who, with a postal card, which costs him one cent, gets a sample upon which the editor pays 2 to 3 cents postage, and which is worth in addition 25 to 50 cents per copy—

"The man's an ignoramus,
Or, lower yet, a scamp,
Who writes for information
And sends no postage stamp."
—*Courier-Journal.*

And meaner he, who claims M.D.,
With pestle, tile and mortar,
Who, by *postals* gets his reading free
From *samples* worth a quarter. W.

